# Polymeric Carriers for Delivery Systems in the Treatment of Chronic Periodontal Disease

**DOI:** 10.3390/polym12071574

**Published:** 2020-07-15

**Authors:** Magdalena Zięba, Paweł Chaber, Khadar Duale, Magdalena Martinka Maksymiak, Maciej Basczok, Marek Kowalczuk, Grazyna Adamus

**Affiliations:** 1 Centre of Polymer and Carbon Materials, Polish Academy of Sciences, 34. M. C. Skłodowska St., 41-800 Zabrze, Poland; pchaber@cmpw-pan.edu.pl (P.C.); kduale@cmpw-pan.edu.pl (K.D.); mmaksymiak@cmpw-pan.edu.pl (M.M.M.); marek.kowalczuk@cmpw-pan.edu.pl (M.K.); 2 Institute of Molecular Biology and Biotechnology, Faculty of Biology, Adam Mickiewicz University, 6 Uniwersytetu Poznańskiego St., 61-614 Poznań, Poland; macbas@amu.edu.pl

**Keywords:** periodontitis (PD), polymers, oral drug delivery systems (DDS), drug release, local drug delivery systems

## Abstract

Periodontitis (PD) is a chronic inflammatory disease of periodontal tissues caused by pathogenic microorganisms and characterized by disruption of the tooth-supporting structures. Conventional drug administration pathways in periodontal disease treatment have many drawbacks such as poor biodistribution, low selectivity of the therapeutic effect, burst release of the drug, and damage to healthy cells. To overcome this limitation, controlled drug delivery systems have been developed as a potential method to address oral infectious disease ailments. The use of drug delivery devices proves to be an excellent auxiliary method in improving the quality and effectiveness in periodontitis treatment, which includes inaccessible periodontal pockets. This review explores the current state of knowledge regarding the applications of various polymer-based delivery systems such as hydrogels, liposomes, micro-, and nanoparticles in the treatment of chronic periodontal disease. Furthermore, to present a more comprehensive understanding of the difficulties concerning the treatment of PD, a brief description of the mechanism and development of the disease is outlined.

## 1. Introduction

Periodontitis (PD) is a localized chronic inflammatory disease of periodontal tissues caused by pathogenic microorganisms and characterized by disruption of the tooth-supporting structures [1]. The inflammation is primarily localized to the gum, but penetrates deeper if left untreated, creating pockets that host anaerobic bacteria which can then lead to further erosion of the tooth attachment and eventually to tooth loss. Periodontitis includes various degenerative and inflammatory states of the tissue surrounding the tooth, e.g., gingival, periodontal ligaments, cementum, and alveolar bone.

**Figure 1:**
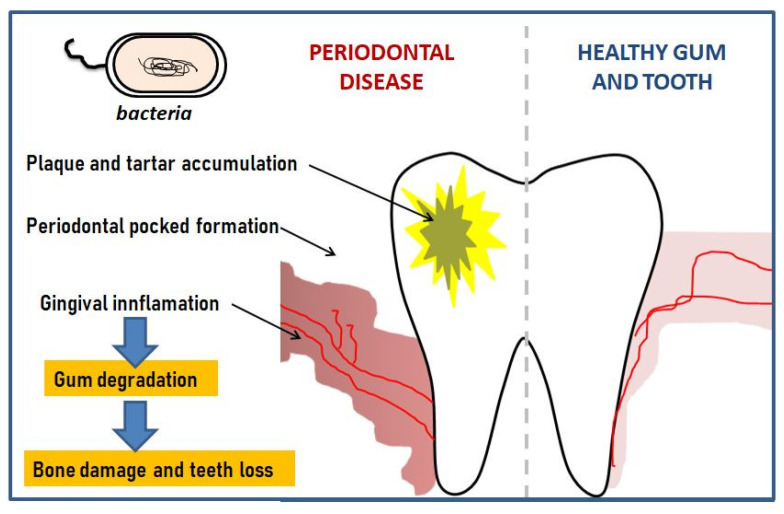
Schematic development of periodontal disease.

According to the World Health Organization report, it is one of the world’s most widespread chronic ailments occurring after the age of 35 [2,3]. Figure 1 shows a schematic depiction of periodontal disease.

The current treatment of periodontal disease involves oral hygiene such as mechanical cleaning (brushing and flossing), together with the use of dentifrices and antibacterial mouthwashes. Non-surgical treatment methods for periodontitis include mechanical scaling and root planning [4]. Additionally, antibiotics, especially tetracyclines, β-lactam, and nitroimidazole antibiotics are mostly used for the treatment of periodontal disease [5].

Conventional drug administration routes are the most popular methods of drug treatment due to certain advantages, such as ease of use and a very high degree of dosage flexibility. This method is used to eliminate or inhibit gingival bacteria flora, decrease inflammation, and help to discontinue bone desorption. However, conventional drug administration pathways also have many drawbacks such as serious side effects, poor biodistribution, low selectivity of the therapeutic effect, burst release of the drug, and damage to healthy cells [6,7]. The efficacy of conventional antimicrobial therapy has often been hampered by the difficulties of achieving and maintaining an appropriate concentration of curative agents at the periodontal site when a low dose of the drug is given. To overcome these limitations, a more effective approach to administering drugs into the periodontal pocket needs to be developed.

Recently, there has been growing interest in controlled drug delivery systems as a whole and as a potential method to address oral infectious disease ailments in particular [8]. Drug delivery systems (DDS) are used vehicles to transport therapeutic agents including antibodies, peptides, vaccines, drugs, and enzymes to a target location and safely achieve the desired therapeutic effect. Thus, the administration and efficacy of these pharmaceutical compounds are improved [9,10]. The effectiveness of medicine has increased with the development of the fabrication of drug delivery carriers, which can administer accurate doses of drugs, minimize side-effects, and improve healthcare treatment of patients [5,11].

Polymers continue to play an important role in the development of drug delivery technology due to their favorable physicochemical properties and offering the possibility of further modifications. Advanced polymer delivery systems have been developed, in recent years thanks to the innovations in chemical engineering technology. Modern advances in drug delivery systems are now based on the design of polymers adapted to specific drugs and designed to perform distinct biological functions. To control the release of drugs and other active substances, a wide range of natural and synthetic origin polymer-carriers have been used. Polymers are potential drug carrier candidates but they must present some characteristics such as efficacy, hydrophilicity, lack of immunogenicity, lack of biological activity, and the presence of functional groups for covalent coupling of drugs or target moieties [12,13,14].

In recent years the field of controlled release drug delivery have been utilizing biodegradable polymers that can biodegrade within the body, such as polylactides (PLA), polyglycolide (PGA), poly(lactide-co-glycolides) (PLGA), polyanhydrides, polyorthoesters, all of which were previously known for other medical applications. The greatest advantage of these biodegradable polymers is that they are broken down into biologically tolerable molecules that are metabolized and removed from the body via normal metabolic pathways [15]. Apart from all these advantages, the use of polymeric carriers also has some limitations. For instance, naturally occurring polymers although highly abundant and biodegradable are difficult to reproduce and purify whereas, empty non-biodegradable polymers delivery devices needed to be removed by surgery after releasing the drug at the targeted site. Moreover, biodegradable materials do produce degradation byproducts that must be tolerated within the biological environment and therefore, both the desired and unwanted by-products must be carefully tested.

Polymer therapeutics include linear or branched polymer chains which act either as the bioactive molecule, e.g., polymeric drug or as the inert carrier to which a drug can be covalently linked, e.g., polymer-drug conjugates, dendrimers, polymeric micelles, nanocapsules, nanospheres, hydrogels, liposomes, etc. However, in order to successfully be used in controlled drug delivery formulations, a polymeric carrier must be chemically inert and free of leachable impurities. In other words, it must have an appropriate physical structure, with minimal undesired aging, and be readily processable [16].

The elaborated periodontal pocket drug delivery devices based on polymers improve the antimicrobial efficacy and demonstrate many consequent clinical benefits. It is worth mentioning that local sustained administration of drugs into the periodontal pockets in the mouth is challenging due to the continuous secretion of saliva. This facilitates for the implant to leave the periodontal pocket easily and it could then be swallowed by the patient [17]. Usage of a local drug delivery system in the treatment of periodontitis allows to achieve and maintain a greater concentration of drug at the diseased area than possible with conventional therapy, preventing the side effects of free-drugs. 

This review aims to provide the reader with an overview of periodontal disease with a particular focus on pathogenesis as well as the applications of polymer-based delivery systems used in the treatment of periodontitis. It narrates current developments and highlights emerging fields, such as liposomes, nanotechnologies, and hydrogels. Treatment of periodontitis with drug delivery systems has attracted increasing attention and the number of publications illustrates the importance of this area.

## 2. The Pathogenesis of Periodontitis Diseases

In healthy individuals the periodontium is mostly colonized by harmless, commensal bacterial communities [18]. A proper balance in their composition prevents the immune system from a potentially detrimental reaction. A dental plaque is defined as a polymicrobial biofilm on the surface of the tooth and tooth root. Presumably, due to poor hygiene or risk factors, excessive dental plaque may accumulate leading to the destruction of periodontal connective tissue and alveolar bone, the disease known as periodontitis [19]. One more important process is connected with this pathological state: a shift in microbial composition from mostly Gram-positive to mostly Gram-negative bacterial species [20,21]. Nevertheless, the mechanism causing the bacterial transition from one state to the other remains to be elucidated and a model implicating Gram-negative bacteria as the sole etiologic factor is rather oversimplistic [21]. 

The gingival epithelium constitutes an interface between the human organism and complex microbial community inhabiting the oral cavity. Hence, it is constantly challenged by numerous bacterial species. Any perturbations must be immediately faced by the immune system. The adaptive immune response requires several days before it may start to combat microbes, therefore innate immunity plays a critical role in the early stages of infection [22]. Pathogen associated molecular patterns (PAMPs) are defined as conserved microbial moieties that trigger the innate arm of the immune system which, in turn, directs adaptive immunity. Usually, they are crucial for microbial survival, thus their evolution rate is strongly inhibited. As PAMPs may serve diverse pathogen structures like lipopolysaccharide (LPS), flagellin, or fimbriae [23,24,25,26]. Pattern recognition receptors (PRRs), located on/in immunologically-relevant cells, are host receptors involved in PAMP recognition and initiation of the inflammatory response. Usually, such a response is beneficial, because it restricts infection and allows for pathogen removal from the host organism, but when the reaction is exaggerated or lasts too long, the inflammation may lead to detrimental effects on the host tissues, including tissue injury, perfusion defects, chronic inflammation state, and sepsis [27].

**Figure 2:**
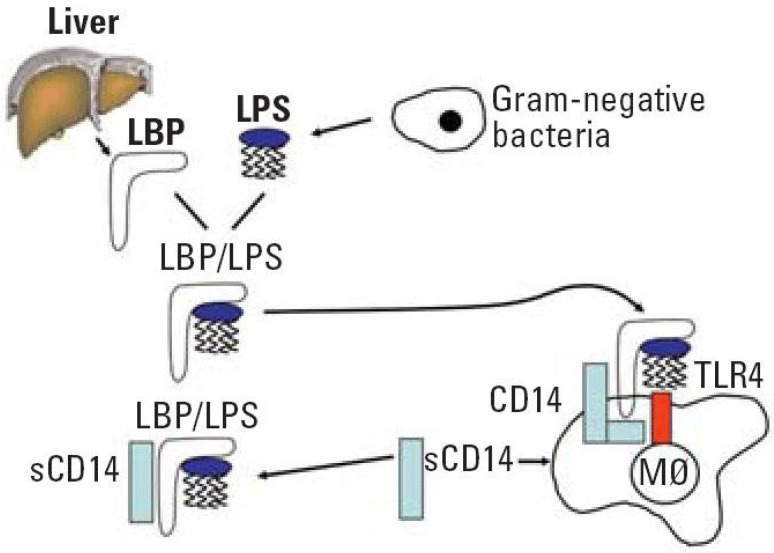
LPS recognition system. Abbreviation: LPS, lipopolysaccharide; LBP, lipopolysaccharide binding protein; CD, cluster of differentiation; sCD14, soluble CD14; TLR4, toll-like receptor 4; M∅ macrophage. Reprinted with permission from [30]. Copyright Environ. Health Perspect., 2006.

A healthy junctional epithelium is rich in many innate mediators [19], and an appropriate expression of interleukin 8 (IL-8), intracellular adhesion molecules (ICAMs) and E-selectin enables neutrophils’ presence within the tissue. Their role is to keep bacteria forming dental plaque under control [28,29]. Since periodontitis is related to a shift from Gram-positive to predominately Gram-negative flora, one of the essential steps for host defense is to correctly detect such a transition. The best characterized PAMP of Gram-negative bacteria is LPS, a major component of the outer membrane. When a pathogen invades the tissues, LPS might be released from the cell surface. Subsequently, it binds to LPS binding protein (LBP), complexes with a soluble or membrane-anchored CD14, and as the LPS-LBP-CD14 complex is transferred to another complex, namely TLR4-MD-2 (Figure 2) [30].

After activation, TLR-4 undergoes oligomerization and triggers downstream molecular cascade which ends with the synthesis of the proinflammatory cytokines and type-1 interferon. The effect of this pathway is then intensification of the immune response and, under standard conditions, infection eradication [25,26]. An interesting finding made by Ren and colleagues [31] is that LBP levels in healthy gingival tissues were significantly higher when compared to tissue with already developed periodontitis. Nonetheless, whether this is a cause or effect of the growing infection is not established. It is worth noting that other PRRs like TLR2 may induce a destructive inflammatory response [32].

**Figure 3:**
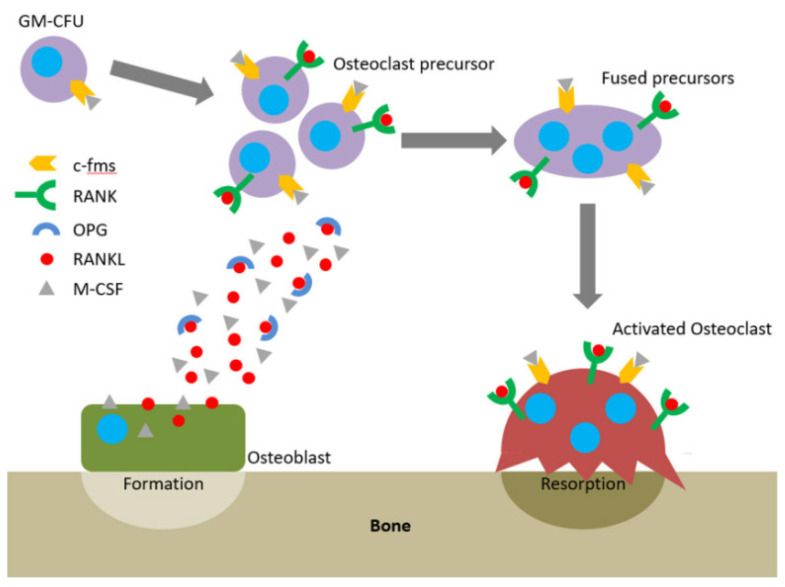
The RANKL/RANK/OPG axis and M-CSF direct osteoclastogenesis and activation. Reprinted with permission from [35]. Copyright Owen and Reilly (2018).

One of the major symptoms of periodontitis is bone loss. Once the immune system fails to limit a local gingival infection, the inflammatory state expands finally reaching alveolar bone and causing severe tissue destruction [33]. In healthy individuals, bone remodeling understood as bone resorption and bone formation is equable [34]. The receptor–activator of nuclear factor-κB ligand (RANKL) /osteoprotegerin (OPG) ratio maintains this equilibrium. OPG functions as a soluble receptor for RANKL, the ligand for the receptor–activator of nuclear factor-κB (RANK) receptor, which is produced by osteoblasts and exists either as a soluble or membrane-anchored protein The interaction between OPG and RANKL prevents RANKL from binding RANK, the receptor RANK present on the osteoclasts and their precursors. RANK binds RANKL and directs precursor cells to differentiate into ones producing enzymes breaking down a bone (Figure 3) [35]. Proinflammatory cytokines such as IL-1β, -6, -11, and -17 and TNF-α lead to greater RANKL expression and drop in the OPG levels [36]. It was proved that in periodontitis the RANKL/OPG balance is disturbed in favor of higher RANKL/OPG ratios [37,38].

However, the bone loss process in periodontitis is more intricate than simple shifts in the RANKL/OPG ratio and involves more signaling molecules like IL-1, IL-6, TNF, or metalloproteinases [39,40,41]. The latter ones are a very important group from the periodontitis pathogenesis point of view. They are protein-degrading enzymes playing a variety of roles, including proteolysis of chemokines, receptors, inhibitors, and collagen, hence significantly influencing inflammation regulatory processes.

Collagen is a basic extracellular matrix component of connective tissue and its destruction results in remarkable tissue impairment [41]. In periodontitis-affected tissues elevated levels of MMP-8 were reported [42] and increased activity of MMP-9 and MMP-13 were observed [43]. It seems that MMP-13 is particularly implicated in the periodontal soft tissue and alveolar bone destruction. MMPs and their detailed role in periodontal tissue breakdown are covered by numerous articles and reviews [41,43].

Although the current model of periodontitis attributes the most severe consequences like tissue defects and teeth loss to be the effects of the oral homeostasis disturbance and overly active immune response [19,41] rather than direct action of periopathogens, some interesting findings have been made in establishing its bacterial aetiology. Two bacterial complexes so-called “orange” and “red” are comprised of species believed to be the major aetiological factors of periodontitis. The orange complex appears first and includes anaerobic or microaerophilic Gram-negative species mainly from *Prevotella*, *Fusobacterium*, and *Campylobacter* genera. As the pathological state progresses, red-complex bacteria—*Tannerella denticola, Tannerella forsythia*, and *Porphyromonas gingivalis* start to multiplicate and colonize gingival tissue [44]. Among them, *P. gingivalis* is the best studied. It uses numerous strategies to circumvent the immune response that allows for a further thriving of surrounding bacterial community ultimately leading to chronic inflammation of periodontal tissues [45]. Examples of its virulence factors are lipid A 1- and 4′-phosphatases that alter LPS into a non-immunogenic form [46], fimbriae promoting colonization of epithelial cells and inducing CXCR4/TLR2 cross-talk which leads to immune response evasion [47] or Hgp44 [48] functioning as bacterial adhesion, to name a few. Although many sides of *P. gingivalis* biology has been unveiled, a lot of aspects remain to be investigated, and it is only the tip of the iceberg. Periodontitis affected tissues are inhabited by plenty of interrelated bacterial species that interact with the host immune mechanisms thus creating extremely complicated signaling and dependencies network. 

Altogether, periodontitis begins with oral homeostasis changes, proceeds with bacterial communities’ expansion to a chronic inflammatory state. Finally, prolonged inflammation severely damages the gingival and alveolar bone tissue integrity.

## 3. Drug Delivery Devices

In general, a drug delivery device or therapeutic system is a vehicle that transports an active agent to a site and releases its contents at a predetermined therapeutic level for a fixed period [48]. The principle of using drug delivery devices in periodontitis treatment dates back to 1979 [49], but the technology has been evolving ever since its first inception and drug delivery devices have emerged as a mainstay in the fight against periodontitis. The most important requirements for a drug delivery device in periodontitis treatments are the delivery of antimicrobial agents in those areas where mechanical scaling instruments cannot access and the release of the level of active agent required throughout the entire treatment period, while at the same time exhibiting cytocompatibility and biocompatibility [50,51]. The device can be placed directly in the periodontal pockets due to the relatively easy accessibility of the pockets; when the drug reaches the pocket, it must remain in place for the pharmacological effect to occur [52]. The drug seeps out of the device into the pocket in many different ways through solute diffusion, polymer matrix swelling, degradation, and erosion of the polymeric material. This occurs simultaneously or at different stages of the delivery process [53]. Diffusion processes are always involved in the drug release of a polymer matrix. For a non-biodegradable polymer matrix, the release rates are concentration gradient dependent and involve either matrix swelling or diffusion. However, for a biodegradable matrix, diffusion is only dominant when erosion is slow since the drug release rate is mainly dictated by the hydrolytic cleavage of polymer chains leading to matrix erosion and drug diffusion. The matrix erosion, in turn, depends on various factors including morphology and processing conditions. Therefore, the drug release rate is dependent on the chemical structure and architectures of the polymeric carrier system, the physicochemical properties of the solutes, but it is also influenced by several other parameters in the surrounding environment, e.g., pH and temperature [54,55]. The kinetics of this release can be explained by the mathematical equations dealing with the drug release methodology [56,57,58]. In many cases, it is desirable to have a controlled delivery system that requires the maintenance of a constant drug concentration for an extended period of time within the therapeutic range without fluctuations in concentration levels [59,60]. 

Various types of drug delivery devices such as fibers, acrylic strips, films, biodegradable gels, solutions, compacts, vesicular systems, paste, nanoparticles, and microparticles have been reported [59,60,61,62]. Earlier devices were nonbiodegradable and required the physical removal of the device from the pocket at the end of the treatment. Currently, drug delivery devices made from natural and biodegradable polymer materials are gaining a lot of attention [55,60,61,62,63,64,65,66,67,68,69]. An additional advantage of using biodegradable material is the absence of the need to revisit a dentist to remove the device. Natural occurring polymers such as chitosan, cellulose, alginate, and synthetic polymers such as poly(ε-caprolactone) (PCL), poly(d,l-lactide) (PLA), poly-(d,l-lactide-co-glycolide) (PLGA), poly- (vinylpyrrolidone) (PVP) and poly(vinyl alcohol) (PVAL) have been extensively investigated [58,67,70,71,72,73,74,75]. Fibers are still a relatively popular form of therapeutic delivery systems and are fabricated mainly by electrospinning. Goodson et al. [60,65] were the first to come up with the idea of drug delivery in the periodontal pocket. They started with monolithic cellulose acetate fibers filled with tetracycline hydrochloride that was tied around the crevice of the pocket and then they proceeded to test other polymers, such as polyethylene, polypropylene, polycaprolactone, polyurethane, cellulose acetate propionate, and ethylene-vinyl acetate copolymer fibers [4,61,62,63,64,65,66,67,68,69]. The researchers have observed that in case polyethylene and polyurethane, the release of the drug was very fast. While polypropylene, polycaprolactone, and cellulose acetate propionate have released rapidly a small percentage of loaded drugs [64]. Their initial studies of fibers, where diffusion-controlled devices and did not produce the desired result due to poor delivery rate control which lead to 95% of the therapeutic agent being released within 2 h, but it remained within therapeutic range in 24 h. However, ethylene-vinyl acetate fibers were found to be an adequate carrier and allowed an in vitro sustained drug delivery of up to 9 days. That led to the first drug delivery device on the market, under the trademark Actisite, which contained 25% tetracycline hydrochloride. Since that time several other local drug delivery devices along with various therapeutic agents have been formulated for the effective management of periodontitis. Different types of DDS, that have been reported in recent years and a comprehensive list of all approved drugs can be found elsewhere [4]. Chitosan is also another polymer that has been investigated extensively. Low molecular weight and highly deacetylated chitosan cross-linked with tetracycline hydrochloride containing poly(vinyl alcohol) (PVA) as the core layer, was used to fabricate of DDS by coaxial electrospinning method in a recent study [58]. The author evaluated physicochemical and biological properties of resulting nonwoven and observed sustained release of tetracycline hydrochloride from the drug delivery device for up to 14 days, while the device was also exhibiting cytocompatibility as well as antibacterial activity against a wide range of periodontal pathogens. Ashri et al. developed mucoadhesive buccal film based on chitosan and polyvinyl pyrrolidone (PVP) as a drug delivery device and observed a sustained release for the anti-inflammatory drug tenoxicam in the buccal cavity [67]. The author suggested that this could provide an alternative treatment for chronic periodontitis since it can provide a means to access hard to reach sides, for normal orally administrated drug, as this adhesive ability offers drug retention to the mucosa or tooth surface for sustained local drug release. 

In another study, Hasnain and coworkers [68] have evaluated natural mucoadhesive polymer extracted from *Dillenia* fruit gum containing 1% w/w aceclofenac. It was observed that the in vitro aceclofenac release from the dental pastes was slowly sustained over 6 h. Since periodontitis involves both bacterial infections and other complications such as inflammation, it may be advantageous to load the drug delivery device with a combination of drugs so that a dual or synergistic effect of the treatment action can be obtained. In a recent study, mucoadhesive thin films consisting of hydroxypropyl methylcellulose (HPMC), and low molecular weight of polyethylene glycol (PEG 400) and Carbopol 917 impregnated with microbicide and anti-inflammatory agents have shown simultaneously in situ delivery of both drugs [69]. A dental floss consisting of hydroxypropyl methylcellulose (HPMC), glycerol, polyethylene glycol 400 (PEG 400), and (polyvinylpyrrolidone-iodine as an antimicrobial agent has been reported as a potential preventive measure against periodontitis disease [4].

## 4. Hydrogels

**Figure 4:**
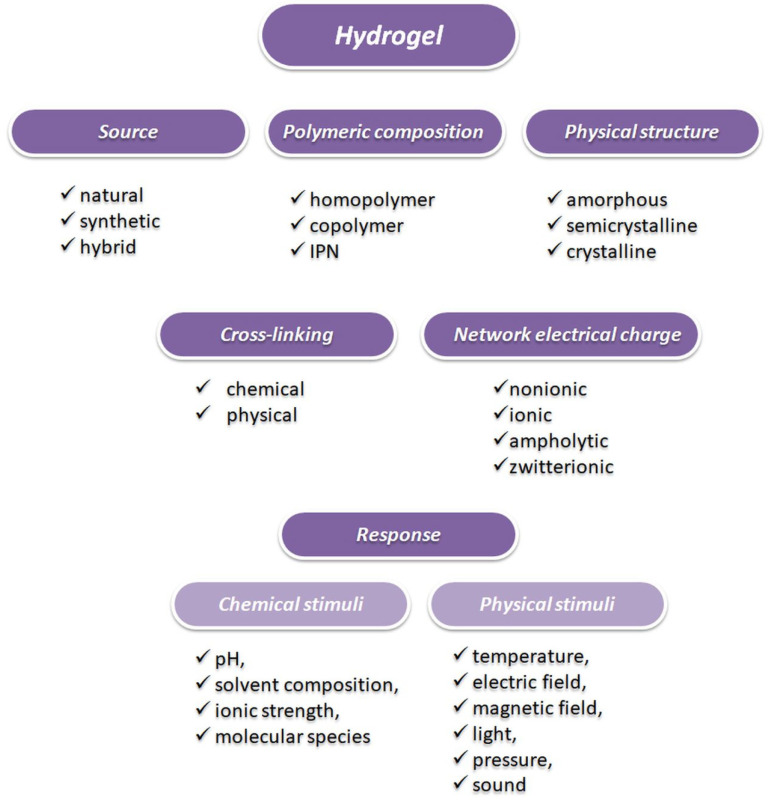
The classification of hydrogels along with all their categories and subcategories.

Hydrogels are three-dimensional, cross-linked networks of water-soluble polymers that contain hydrophilic groups such as hydroxyl or amide groups in their structure. Polymer networks can be formed by cross-linking [76]. The cross-linking reaction involves the formation of covalent bonds, or the physical interactions, such as molecular entanglement, ionic interaction, and hydrogen bonding, amongst the polymeric chains [77]. Hydrogels can swell (absorb a large volume of water) and then shrink for controlled drug release. The high content of water (about 70–99%) in hydrogels provides good biocompatibility, biodegradability, low cytotoxicity, moreover, they pose physicochemical similarities to the native extracellular matrix (ECM) [78,79,80,81]. Besides, hydrogels demonstrate low elastic surface tension with water and biological fluids, which makes them tolerable [81,82]. In the literature, several classifications of hydrogels have been reported, which is due to their different properties. Hydrogels can be differentiated based on the monomer origin (natural, synthetic, and hybrid), the method of preparation, or the presence of electric charge. Due to their chemical and physical properties, hydrogels can respond to different types of stimuli including physical, and chemical stimuli. The properties of hydrogels can be designed and tailored for the special requirements in the different applications. Figure 4 shows a classification of hydrogels along with all categories and subcategories [82,83].

It is worth mentioning that the kinetics of release of bioactive substances, drugs, etc. from the hydrogel matrix depends on the composition of the hydrogel (a type of polymer, drug or additives), geometry (such as size and shape), preparation technique, environmental conditions during drug release as well as the physical and chemical inter-reaction between the hydrogel and bioactive substances can affect the release kinetics of these bioactive substances [76].

The increasing demand for new and more effective polymer-based drug carriers for the medical field has led to the development of materials with tailored-made characteristics for special applications. Consideration of the formulation of periodontal drug delivery systems requires the knowledge of anatomy, physiology, biochemistry, and microbiological etiology of the periodontal region [84]. Mechanical properties such as hardness, compressibility, adhesiveness, and cohesiveness should be considered when the DDS is designed [85].

The first three parameters characterize the obtained system in terms of ease of application and retention capacity of a carrier in the periodontal pocket. Furthermore, the cohesiveness is a factor that will influence product performance. The hardness refers to a deformation ability that spreads between DDS and oral mucosa. Also, bioadhesion is an important factor, which should be considered in the development of local drug delivery systems. This term means attachment of a drug carrier to a specific biological location for prolonged periods of time, which can be epithelial tissue or the mucous coat on the surface of a tissue. In the second case, the phenomenon is called as mucoadhesion. Application of bioadhesive polymers in the treatment of periodontal disease could improve and enhance the bioavailability of drugs, offer prolonged contact at the site of action, allow an intensified contact with the epithelial barrier, and decreasing the frequency of dosage [86,87]. Taking into consideration the future application of the system, syringeability and injectability should be evaluated. Nowadays, syringeability/injectable hydrogels have received more attention due to the fact that they allow the intrusive surgical procedure, the possibility of the tailored shape in real-time, and also simple and easy handling [88].

**Figure 5:**
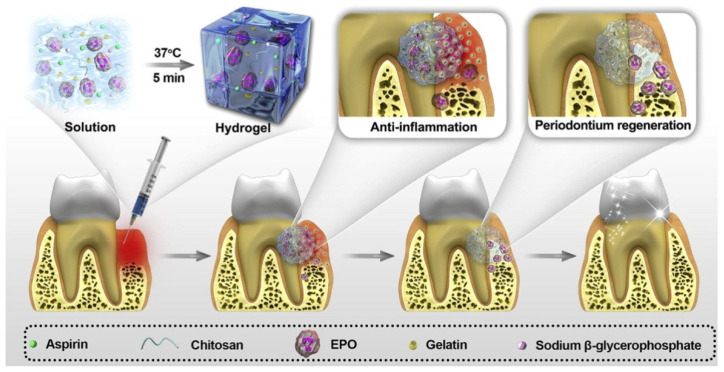
Graphical illustration of the preparation and application of the CS/β GP/gelatin hydrogels. Reprinted with permission from [90]. Copyright Elsevier (2019).

In recent years, the injectable thermosensitive hydrogels are gaining more attention for the treatment of often unapproachable periodontal pockets. In 2019, Xu et al. developed injectable and thermosensitive hydrogels loaded with aspirin and erythropoietin, which can continuously release the drug to exert pharmacological effects of anti-inflammation and tissue regeneration, respectively (Figure 5) [89]. Their hydrogels were mainly manufactured from chitosan (CS) and β-sodium glycerophosphate with the addition of gelatin into the hydrogel matrix as a crosslinking agent, which works through electrostatic interaction to minimize the gelation time. Chitosan is a soluble polysaccharide which consists of N-acetyl D-glucosamine and D-glucosamine units. Also, CS is a natural biopolymer that shows antioxidant activity and antimicrobial activity against various microorganisms such as Gram-positive and Gram-negative bacteria, filamentous fungi, and yeast [90]. β-Glycerophosphate (GP) is a protein phosphatase inhibitor that acts as a phosphate group donor in matrix mineralization research and promotes bone matrix mineralization [91]. The addition of β-GP to CS aims to reduce the polarity of the chitosan chain and increase hydrophobic interactions, which leads to the production of thermosensitive hydrogels [92]. The sol-gel transition occurred at 37 ℃. The release profile of drugs from the hydrogels showed that the release amounts of aspirin and erythropoietin in the first 8 days were approximately 94.2% and 83.4%, respectively. The authors did not observe any obvious inflammatory responses, i.e., local redness, swelling, abscess, or necrosis, indicating no toxicity of the hydrogels in the in vivo test. Also, no cyclooxygenase-2 (COX-2) positive cells were found around the hydrogels, which also suggests no inflammation and good biocompatibility of the hydrogels. They have demonstrated that the obtained aspirin loaded hydrogels have an anti-inflammatory effect and provide a favorable microenvironment for periodontium regeneration.

Ji et al. have prepared a thermosensitive hydrogel based on chitosan (CS), quaternized chitosan (HTCC), and β-glycerophosphate (GP) loaded with 0.1% w/w chlorhexidine (CS–HTCC/GP) [93]. The hydrogel has been introduced in the liquid form, and the gelation took up to 6 min when the temperature reached 37 ℃. They have been demonstrated that the presence of modified quaternized chitosan inside the hydrogel increased antimicrobial activity compared to non-modified chitosan. Nonetheless, the release rate of chlorhexidine (CHX) was slower with modified chitosan CS-HTCC/GP than chitosan and glycerophosphate (CS/GP) system. After 18 h the amount of released CHX in artificial saliva was 68% and 85%, respectively [94]. Also, thermoresponsive hydrogels CS-HTCC/GP with 0.1% loaded chlorhexidine (CHX) showed an excellent antibacterial effect against bacterial antigens such as *Porphyromonas gingivalis, Prevotella intermedia,* and *Actinobacillus actinomycetemcomitans.*

**Figure 6:**
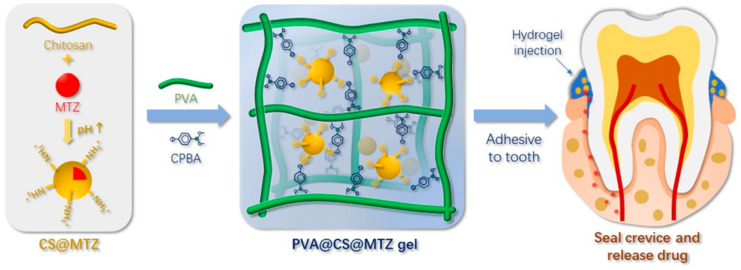
Schematic illustration of the preparation of chitosan-loaded metronidazole microcapsules (CS@MTZ) and the cross-linking mechanism with CPBA-grafted PVA to obtain injectable hydrogel. Reprinted with permission from [17]. Copyright ACS Publications (2019).

Dong and co-workers have prepared an injectable hydrogel system based on poly(vinyl alcohol) (PVA) with metronidazole (MTZ) microcapsules (CS-MTZ) incorporated in chitosan (CS) (Figure 6) [17]. To obtain a crosslinked hydrogel, they have used a dually dynamic crosslinking strategy with 4-carboxyphenylboronic acid (CPBA). This strategy relies on used a small and rigid molecule with two functional groups, like CPBA, which to react with a polymer, i.e., poly(vinyl alcohol) (PVA), to form borate bonds and ionic interactions to bridge the polymer chains in the presence of metronidazole microcapsules (CS-MTZ).

The hydrogel exhibited adhesive properties under wet conditions, which is very important for the formulation of devices with prolonged residence in periodontal pockets. The hydrogel matrix as well as the capsule successfully slowed down the MTZ release at neutral pH. The release kinetics became faster under weakly acidic pH conditions because of the dissolution of the chitosan.

The lower concentration (about 1%) of CS-MTZ microcapsules in the matrix resulted in faster release of the drug from the hydrogel, as compared to the hydrogel containing 2% of the particles due to a less crosslinked hydrogel network. The studies confirmed the bacteriostatic effectiveness of the obtained hydrogel formulation. The PVA-CS-MTZ hydrogel achieved continuous bacteriostatic and sterilizing effects for two weeks in the absence of an organic medium, which confirmed the efficiency of the system on the controlled release of the water-soluble drug. Also, in vivo studies have proven the safety, biocompatibility, and antibacterial efficacy of PVA-CS-MTZ hydrogels [94]. The average probing depth of the periodontal pocket was significantly reduced after using a hydrogel preparation compared to a commercially available drug or no treatment.

Recently, in situ gel formulation for local delivery moxifloxacin hydrochloride in periodontal disease has been developed by Swain et al. [95]. The gel formulations were prepared using thermally-sensitive (Poloxamer 407), ion-sensitive (Gellan gum), and pH-sensitive (Carbopol 934P) polymers. Poloxamer 407 is a triblock copolymer consisting of a central hydrophobic block of polypropylene glycol terminated by two hydrophilic blocks of polyethylene glycol (PEG). Gellan gum (GG) is a linear, anionic exopolysaccharide produced by a non-pathogenic strain of *Sphinogomonas elodea*. The chain of GG consists of four repeating units of carbohydrate, which include α-l-rhamnose, β-d-glucose, and β-d-glucuronate, in a 1:2:1 molar ratio [96], whereas Carbopol 934P is a mucoadhesive polymer that has been investigated as a useful adjuvant for bioadhesive drug delivery systems [97]. Carbopol 934P belongs to a polyacrylic acid resin family known as the carbomers, which is a suitable candidate for usage in drug delivery systems. Carbopol 934P remains in solution form at acidic pH and forms a gel at alkaline pH. The obtained formulations remain in liquid form at non-physiologic conditions (10–25 °C) and form a gel at 37 °C. Among the various formulations optimized batches, the best results were obtained for the system, which contains 19.072% *w/v* Poloxamer 407 and 0.245% *w/v* Gellan Gum, which showed a desired gelation temperature at 37 °C and a gelling time of 102 s. After 1 h the drug release amounted to 23%, whereas the time required to release 90% of the moxifloxacin hydrochloride from the sample was 9.1 h. The authors have also evaluated the mechanical properties, i.e., hardness, compressibility, adhesiveness and cohesiveness, and elasticity with the aid of instrumental texture profile analysis (TPA). The research has demonstrated that the hardness value of the optimized formulation was found to be 190 g and the adhesiveness was 0.60 mJ. They have proven that the hardness of the formulation increases as the concentration of polymer increases. In vitro antibacterial activity was determined by measuring the zone of inhibition against selected strain *Staphylococcus aureus* and *Escherichia coli*. The study indicated that moxifloxacin hydrochloride in the gel-forming system retained its antimicrobial activity. 

The hydrogels based on methylcellulose (MC) with tamarind seed xyloglucan (TSX) to in situ delivery of metronidazole (MTZ) have been developed in 2020 by Hirun and coworkers [98]. Methylcellulose is cellulose modified at the C2, C3, and C6 positions of the anhydroglucose repeating unit which obtained by substitution of hydroxyl groups with methyl groups. Currently, methylcellulose is extensively used in medicine and cosmetics as well as in the food industry. MC is a water-soluble polymer at low temperature, whereas the sol-gel transition temperature is about 40–50 ℃. The tamarind seed xyloglucan is composed of mainly three sugars units, i.e., glucose, galactose, and xylose with a molar ratio of 3:1:2. A very important feature is that both MC and TSX polymers are biodegradable and non-toxic biomaterials. The researchers have prepared hydrogels with different concentrations of MC. They have obtained three hydrogels: 6% *w*/*v* of MC with 1.5% *w*/*v* of TSX (labeled as 6MC-TSX), 7% of MC (labeled as 7MC) and 7% *w*/*v* MC with 1.5% *w*/*v* TSX (labeled as 7MC-TSX). Obtained MC-TSX hydrogels are solutions at temperature 25 ℃, and the sol-gel transition occurs at 36 ℃, 36 ℃, and 34 ℃, respectively. Additionally, the authors have observed that the viscosity increased with increasing MC concentration. Higher viscosity was observed to samples with the addition of TSX. Besides, the researchers have observed good mucoadhesive and injectability properties for samples which are a very important factor in treating dental diseases. It was shown that the release profile of MZT was longer for 7MC/TSX than TSX and 7MC hydrogels and was comparable to a commercial preparation (Metrogyl gel).

Mou et al. [99] have designed a nano-hydrogel which exhibited interesting properties such as pH-responsiveness, wide antimicrobial spectrum, sustained release, tissue-repairing and adhesive properties. Such hydrogel was prepared from the polyacrylic acid (Carbapol 940) with additive albumin microspheres-loaded minocycline and zinc oxide nanoparticles (Mino-ZnO@Alb). The serum albumin microspheres contained 0.06% of minocycline and 0.025% of ZnO NPs. The average size of the obtained spheres was 139 ± 0.42 nm. The gingival redness and swelling have disappeared after treatment with the hydrogel. Furthermore, the probing pocket depth and the bleeding index decreased significantly.

## 5. Liposomes

The name liposome is derived from two Greek words: ‘lipos’ meaning fat and ‘soma’ meaning body [100]. Liposomes are small artificial vesicles of spherical shape that can be formed from cholesterol and natural non-toxic phospholipids [101]. Liposomes as a carrier for target drug delivery were first introduced by Gregoriadis [102]. Liposomes are very promising systems for drug delivery due to their non-toxicity, biodegradability, biocompatibility, and ability to act as carriers for hydrophobic, hydrophilic, and amphiphilic molecules [103]. They consist of single or multiple lipid bilayers created by hydrophilic and hydrophobic interactions with the aqueous phase. Properties of liposomes can differ distinctly because of their size, lipid composition, surface charge, and the method of preparation [104,105].

**Figure 7:**
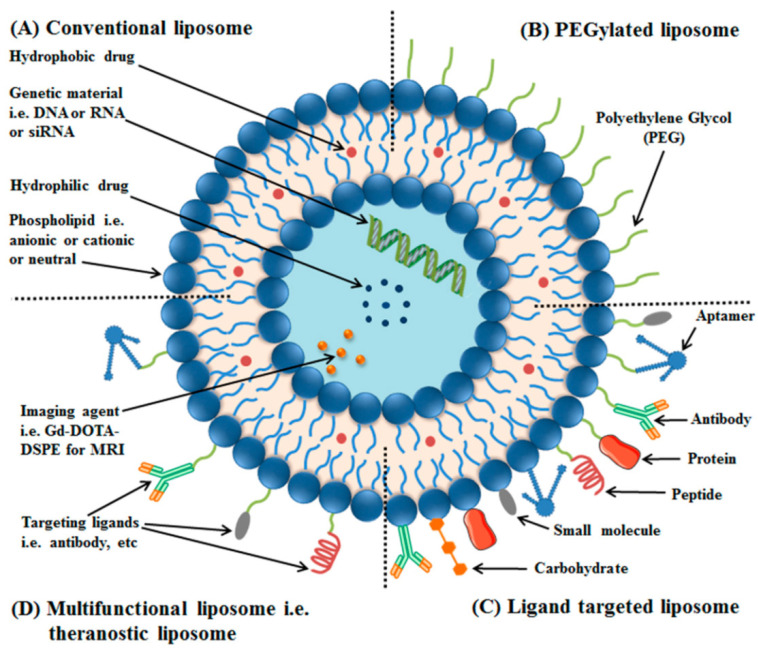
Schematic illustration of liposomes, which are made of phospholipids (**A**); PEGylated/stealth liposomes contain a layer of polyethylene glycol (PEG) at the surface of liposomes (**B**); targeted liposomes contain a specific targeting ligand to target a cancer site (**C**); and multifunctional such as theranostic liposomes, which can be used for diagnosis and treatment of solid tumors (**D**). Reprinted with permission from [106]. Copyright MDPI (2018).

The liposomes can be surface functionalized by coating with a hydrophilic polymer, i.e., polyethylene glycol (PEG) (Figure 7). Such surface-modified liposomes have been termed as PEGylated or stealth liposomes. This modification extends liposomal circulation half-life in vivo by reducing clearance, immune recognition, and the non-specific absorption of serum proteins. Liposomes can also be modified with a suitable targeting ligand with a single or a variety of surface engineering techniques, i.e., proteins, peptides, antibodies, or their fragments, aptamers, small molecules, etc. (Figure 7) [106].

Di Turp et al. have developed liposome-based delivery systems of submicron dimensions which can diffuse into the dentinal tubules [107]. The liposomes were prepared by hydration of a lyophilized preliposomal formulation consisting of the following components are l-α-phosphatidylcholine β-oleoyl-γ-palmitoyl (POPC), l-α-phosphatidylcholine–d,l-glycerol dioleoyl (PGDO) and cholesterol (CHOL). The obtained vesicles were stabilized with polyethylene glycol (PEG-8000). Magnetic nanoparticles were then synthesized inside the vesicles by chemical reaction of FeCl_2_, FeCl_3_, and NH_3_. Vesicle resizing was achieved by sonication or extrusion, however, dynamic light scattering (DLS) analysis showed that the best dimensions were achieved with the extrusion kit. These vesicles exhibited an average diameter of 132.2 nm with a polydispersity index Ð = 0.127. Submicrometer vesicles achieved by the extrusion method can penetrate dentinal tubules. The penetration dentinal tubules can be more extensive when an external magnetic field is applied which accelerates their movement within the duct. 

In another paper, Liu et al. developed nanoliposomes as carriers of an anti-inflammatory action drug—minocycline hydrochloride [108]. The nanoliposomes consist of hydrogenated soy phosphatidylcholine and cholesterol (in a 2:1 molar ratio) and have been prepared via extrusion. Inhibition of mouse macrophage proliferation have been observed and anti-inflammatory effects were achieved by suppressing tumor necrosis factor mRNA expression α (TNF-α) at a reduced dose. The study has shown that nanoliposomes loaded with 2% minocycline hydrochloride had significantly stronger and longer inhibition effect on LPS-stimulated TNF-α secretion of macrophages cell than commercially available dental ointment—Periocline—and 2% minocycline hydrochloride solution.

Although a large number of scientific articles on the formulation and use of liposomes in the treatment of various types of diseases have been published in the literature, only a very few of these are related to the use of liposomes as carriers in the treatment of periodontitis.

## 6. Micro- and Nanoparticles 

Microparticles and nanoparticles are also known as microspheres and nanospheres, respectively. They are small spherical polymeric particles with different diameters. Microparticle diameters vary in the micrometer range (typically from 1 µm to 1000 µm). Nanoparticles are considered as the end product of a wide range of chemical, physical, or magnetic interactions and biological processes leading to very small particles within the range of 1–100 nm, according to new definitions by the U.S. Food and Drug Administration [109,110,111]. The particle size and surface characteristics can be easily changed to suit the requirements for the delivery of the drugs [110]. The highest potential for in vivo applications shows intermediate-size polymeric nanoparticles (20–100 nm) due to their ability to circulate in the bloodstream for long periods of time [111]. Polymeric microparticles are usually formed by a polymer matrix in which a smaller amount of an active compound can be immobilized [88,112].

Micro- and nanospheres can be produced from natural or synthetic polymers, which can be biodegradable or nondegradable. The polymers utilized for microencapsulation include biodegradable polymers such as poly(d,l-lactide-co-glycolide) (PLGA), poly(lactide) (PLA), poly-hydroxyalkanoates (PHA), or natural polymers like chitosan, pectin, hyaluronic acid, as well as polyethylene glycol (PEG). There are several methods for manufacturing the micro- and nanoparticles including emulsification-solvent evaporation, coacervation, spray drying, electrospraying, and phase separation [3,113]. One of the crucial factors affecting the properties of microspheres (such as physiochemical and mechanical properties, mechanism, and rate of biodegradation, toxicity), is the type of polymer used which determines the time and the release rate of therapeutic agents in the targeted site. Controlled release drug delivery systems based on micro and nanospheres have been gaining increased interest in recent years.

A recent study by Lu et al. have developed the microspheres of dextran/poly(d,l-lactide-co-glycolide) (PLGA) loaded with interleukin 1 receptor antagonist (IL-1ra) and then have evaluated the obtained system for the physicochemical and anti-inflammatory properties [114]. Dextran is a natural and biodegradable exopolysaccharide consisting of α-1,6-linked d-glucopyranose. Dextran is soluble in water and organic solvents [115]. The authors used poly(d,l-lactide-co-glycolide) because it is a well-established biodegradable polymer used with great success in the medical field. PLGA is a copolymer d,l-lactide and glycolide obtained via ring-opening polymerization of cyclic dimers of lactic acid (lactide) and glycolic acid (glycolide). In this study have been described two methods to obtain loaded microspheres. The first one chosen method by authors was the water-in-oil-in-water method (W/O/W) for IL-1ra-loaded PLGA microspheres. The second method for fabricating microspheres L-1ra-loaded dextran/PLGA was the solid-oil-water method (S/O/W). The average particle sizes of microparticles were 12.76 ± 4.89 μm and 7.25 ± 4.35 μm, respectively. Also, they have evaluated both encapsulation and loading efficiency with the aid of an ELISA kit. The results have shown that the microspheres have good biocompatibility and can effectively inhibit the gene expression of pro-inflammatory factors induced by interleukin 1β (IL-1β) in human gingival fibroblasts.

In a more recent study, a tunable and injectable local drug delivery system for periodontal applications have been developed by Wang et al. [116]. Microspheres have been obtained from carboxyl-terminated or ester-capped poly (d,l-lactide-co-glycolide) via an electrospraying method Hence, the obtained systems exhibited a round shape and smooth surface. Doxycycline (DOX) and lipoxin A4 (LXA4) were chosen as therapeutic model drugs. The obtained drug delivery system exhibited good injectability, long-term structural stability, and no obvious in vivo inflammatory response, which is important in such an application.

Another example of the spheres was presented by Novac and coworkers. They have developed interesting controlled release systems for ciprofloxacin based on gellan gum derivatives containing quaternary ammonium groups [117]. Grafting of *N*-(3-chloro-2-hydroxypropyl)trimethylammonium chloride onto primary hydroxyl groups of gellan gum by nucleophilic substitution, in the presence of alkali, under specific reaction conditions using various gellan gum/*N*-(3-chloro-2-hydroxypropyl)-trimethylammonium chloride molar ratios was used to obtain quaternized gellan gum derivatives. The obtained products have been characterized by ^1^H-NMR and FT-IR. The obtained particles have been evaluated by the antibacterial test which confirmed the efficiency of the quaterized gellan gum particles loaded with ciprofloxacin against Gram-negative bacteria compared with Gram-positive bacteria. Also, the results have been obtained due to the electrostatic interactions that may take place between the quaternary ammonium moieties on the particles’ surface and the negatively charged groups of microbial cell membranes. In vitro, transdermal release tests confirmed that ciprofloxacin was released in a controlled manner from these particles for up to 24 h, therefore, proving their potential usefulness as controlled release systems of antibacterial substances.

Nanoparticles also represent a promising strategy for selective and controlled drug delivery into cells and tissues, inducing a uniform drug concentration at the periodontal inflammation sites and thus reducing bleeding. A promising method of preparation of nanoparticles (NPs), which could be used as drug carriers for the treatment of periodontal disease and consisting of poly(d,l-lactide-co-glycolide) (PLGA), poly(d,l-lactide) (PLA) and cellulose acetate phthalate (CAP) and poly(vinyl alcohol) (PVAL) as a stabilizer, was prepared with different amounts of triclosan (TCS) by Piñón-Segundo et al. [118]. These polymeric submicron particles were prepared using the emulsification—diffusion technique and were less than 500 nm in size. A fast release of TCS from these NPs was then detected. A preliminary in vivo study in dogs with induced periodontal defects proposed that TCS-loaded NPs penetrate through the junctional epithelium.

A new strategy for periodontal disease treatment was suggested by Osorio et al. [70]. They elaborated zinc or calcium loaded PolymP-nActive NPs composed of connected covalently 2-hydroxyethyl methacrylate, ethylene glycol dimethacrylate, and methacrylic acid. To obtain the respective nanoparticles, they used the polymerization precipitation (surfactant-free) in a non-solvent medium technique. Non-loaded nanoparticles were proved to be nontoxic in all the assays, and calcium and zinc-loaded particles showed a dose-dependent but very low cytotoxic effect. Therefore, the nanoparticles presented in this study may be considered as a promising tool for a therapeutic approach to periodontal regeneration. 

Beg et al. have described the methodical development of a new in situ gel formulation containing poly(lactide-co-glycolide) PLGA nanoparticles for targeted delivery of moxifloxacin (MOX) against bacterial periodontitis [71,119,120]. MOX-PLGA nanoparticle-loaded in situ gel formulation was prepared by the cold mixing method. The delivery system presented had extended drug release and enhanced retention ability in the periodontal site based on in vivo histopathological studies and in vivo gamma scintigraphy studies. The authors claim that the achieved results of this temperature-responsive formulation can be successfully extrapolated to other antimicrobials commonly prescribed for the treatment of periodontitis. 

A polymeric nanoparticle drug delivery system comprising of 1-butyl-3-methylimidazolium hexafluorophosphate ([BMIM][PF6]) ionic liquid (IL), incorporated into a base material consisting of poly(d,l-lactide-co-glycolide) (PLGA), polyvinyl caprolactam (PVCL), polyvinyl acetate (PVA), polyethylene glycol graft copolymer (Soluplus^®^, Sol) have been reported [121]. It was observed that the ionic liquid increases the retention ability of the polymeric nanoparticles even in the presence of saliva. Moreover, the IL also exhibited anti-microbial activity by causing cracking in the bacterial cell wall and cytoplasmic membrane, therefore the polymeric nanoparticle showed high antibacterial activity even without any impregnated drugs. 

Several insightful works covering different aspects of organic and inorganic nanoparticles syntheses and application studies have been published in the past few years [99,121]. The work by Mota et al. proposes a combination of chitosan (CS) with bioactive glass nanoparticles (BG-NPs) in order to obtain a novel guided tissue and bone regeneration membrane. The studies on CS/BG-NP considered possible application as guided tissue regeneration (GTR) membranes and for guided bone regeneration (GBR), provided by the addition of BG-NPs to the polymer, and promoting the possibility of periodontal regeneration. The authors proposed the use of CS/BG-NP composite membranes as barrier membranes to prevent the invasion of periodontal defects by soft tissues, since these membranes did not show early degradation and were non-cytotoxic. Human periodontal ligament cells and human bone marrow stromal cells have been used to carry out the respective biological tests. Additionally, CS/BG-NP composite membranes promoted cell metabolic activity and mineralization [122].

## 7. Conclusions and Future Perspectives

Conventional treatment of periodontal disease is long-term, and the currently available therapeutic substances are insufficiently effective and highly toxic to humans. Likewise, there are frequent side effects of drug use like poor biodistribution, low selectivity of the therapeutic effect, burst release of the drug, and damage to healthy cells. The above side effects are generally caused by incorrect drug dosing. From the medical point of view, an interesting approach is the use of controlled drug release systems, in which dynamic development has been observed in recent years in treating periodontal disease. The solution is modern drug formulations that allow increased bioavailability (solubility, stability in vivo, membrane permeability), and improve the overall therapeutic effectiveness Polymeric drug carriers compared with other carriers have shown attractive properties such as biodegradability, tissue biocompatibility, and effectiveness for encapsulation of different antibiotics. In the literature, a large number of scientific articles on the formation of controlled release systems of antibacterial substances with the use of synthetic and natural polymers such as poly(d,l-lactide-co-glycolide), polylactide or chitosan has been observed. Various types of DDS have been developed as potential drug vehicles such as nanoparticles, hydrogels, liposomes, etc., which showed effective antibacterial action against various bacterial strains. Local drug delivery systems have been developed to provide controlled and sustained release of antibacterial agents in the vicinity environment of the inflammation site.

With the continuous development in the pharmaceutical fields, there is no doubt that drug delivery systems based on biopolymers can vastly improve the treatment of various infections and diseases. Targeted pharmacological therapy for periodontitis is an active area of ongoing research that needs to be further developed to evolve better therapeutic strategies and improve the patient experience. There is still a high demand for new, effective drug delivery systems for the treatment of periodontal disease, and further studies on this area are therefore highly anticipated.

The present review has demonstrated many different polymer-based drug delivery systems that can be potentially used to fight against periodontitis diseases. However, there are some challenges in using polymers as carriers in DDS for the treatment of periodontal disease, which should be resolved in the future. The first of them is related to a portfolio of appropriate polymers for application in DDS for periodontal treatment. To meet these requirements of effective drug delivery systems, the tailor-made polymers with relatively complex structures are increasingly used, which is associated with the complexity of their synthesis. That is why it is important to develop procedures that allow the synthesis of such polymeric carriers with high homogeneity (low dispersity) in a repeatable manner. The second direction, which can improve the comfort of patients can be the optimization of the size of devices used for local treatment. In this case, a promising effect can give the development of nanoscale intrapocket devices which through delivery even of a low dose of the drug, enable efficient treatment. This idea, however, requires the use of both suitable polymer materials, a method of processing them as well as the method of the preparation of the respective medical devices.

Currently, different antibiotics such as tetracyclines including doxycycline and minocycline, metronidazole, and chlorhexidine are used for local delivery, however, these are known to cause undesirable side effects, such as stomatitis, onycholysis, skin discoloration and decreased blood platelets, just to name a few. Therefore, it is important to implement a new trend utilizing natural products (i.e., drugs possessing antibiotic properties) in such systems. It is also expected that cooperation among polymer chemists, biologists, and dental specialists in the forthcoming future as well as results of their studies significantly broaden the knowledge of the possibility of using polymeric carriers of herbal active substances in the field of medical science, particularly in the field of periodontal disease treatment.
